# Evidence that Osteocytes in Autogenous Bone Fragments can Repair Disrupted Canalicular Networks and Connect with Osteocytes in *de novo* Formed Bone on the Fragment Surface

**DOI:** 10.1007/s00223-017-0283-2

**Published:** 2017-05-10

**Authors:** Furqan A. Shah, Anders Palmquist

**Affiliations:** 1 Department of Biomaterials, Institute of Clinical Sciences, Sahlgrenska Academy at University of Gothenburg, Göteborg, Sweden; 2 BIOMATCELL VINN Excellence Center of Biomaterials and Cell Therapy, Göteborg, Sweden

**Keywords:** Autogenous bone, Early implant healing, Osteocyte, Scanning electron microscopy

## Abstract

Autogenous bone fragments generated during surgery (e.g. implant site preparation) accelerate bone formation by the release of a large variety of growth factors from the extracellular matrix and the cells contained within. Osteocytes, whether viable or apoptotic, within such fragments are able to recruit osteoclasts to a site of bone remodelling. Here, using correlative scanning electron microscopy, we provide compelling evidence that at one week healing in the Sprague Dawley rat tibia, following surgery (and/or the placement of a bone-anchored implant), autogenous bone fragments support bone formation on their surface. Furthermore, osteocytes within the autogenous fragments are frequently able to repair the disrupted canalicular networks and appear to connect with osteocytes (or osteoblastic-osteocytes) in the de novo formed bone on the surface of the fragment.

## Introduction

Autogenous bone, still considered the gold standard for most bone graft applications, osseointegrates with the surrounding bone and undergoes vascularisation [1], and is believed to provide optimal osteoconductive, osteoinductive, and osteogenic properties [2]. A number of growth factors affecting bone formation and resorption may be released from the extracellular matrix and cells (e.g. osteoblasts) in autogenous bone grafts including transforming growth factor beta (TGF-β) and bone morphogenetic protein (BMP), fibroblast growth factor (FGF), insulin-like growth factor (IGF), platelet-derived growth factor (PDGF) [3], vascular endothelial growth factor (VEGF), receptor activator of nuclear factor kappa-β ligand (RANKL), and osteoprotegerin (OPG) [4]. Osteoclasts on the surface of autogenous fresh rib grafts are observed in close contact with the cytoplasmic processes of apparently viable osteocytes within the graft [5]. Viable osteocytes have also been reported within a microvascular fibula flap used in combination with an additively manufactured osteosynthesis plate after 33 months in the human mandible [6]. Both viable and apoptotic osteocytes can recruit osteoclasts to sites of bone remodelling [7]. In the context of peri-implant healing, it is claimed that autogenous bone fragments accelerate bone formation and offer the possibility of earlier implant loading [8]; however, bone fragments generated during implant site preparation are believed to be devoid of osteocytes [9, 10]. While it may be true that the destructive nature of the drilling process could render osteocytes non-viable, we report compelling evidence that such an assumption may not hold true in all cases. Here, we demonstrate that osteocytes in autogenous bone fragments can potentially restore disrupted canalicular networks and connect with osteocytes in the bone formed on the surface of such fragments.

## Materials and Methods

Bone-implant blocks embedded in LR White resin (London Resin Company, UK) were obtained from in vivo animal experiments conducted previously in our group. All specimens represented an early healing stage, i.e. 6–7 days, following the placement of commercially pure (Grade 4) titanium (cp-Ti) implants with and without different commonly applied surface modifications in Sprague Dawley rat tibia. Resin embedded bone-implant blocks were polished using 400–4000 grit SiC paper and examined in a Quanta 200 environmental SEM (FEI Company, The Netherlands) operated in the backscattered electron (BSE) mode at 20 kV accelerating voltage, 0.5 Torr water vapour pressure. Elemental analysis was performed using energy dispersive X-ray spectroscopy (INCA EDX system, Oxford Instruments GmbH, Wiesbaden, Germany) performed at 15–20 kV accelerating voltage, 10 mm working distance, 40 µm aperture size, and 0–10 keV spectral energy range. Elemental maps for the Kα X-ray emission lines for calcium (~3.691 keV), phosphorus (~2.013 keV), and carbon (~0.277 keV) are shown. The osteocyte lacuno-canalicular network was exposed by resin cast etching for direct visualisation [11]. Briefly, the polished resin embedded bone-implant blocks were sequentially immersed in 9% ortho-phosphoric acid and 5% sodium hypochlorite. After overnight drying, the samples were Au sputter-coated (10 nm), and examined in an Ultra 55 FEG SEM (Leo Electron Microscopy Ltd, UK) operated in the secondary electron (SE) mode at 5 kV accelerating voltage and 5 mm working distance.

## Results

**Fig. 1:**
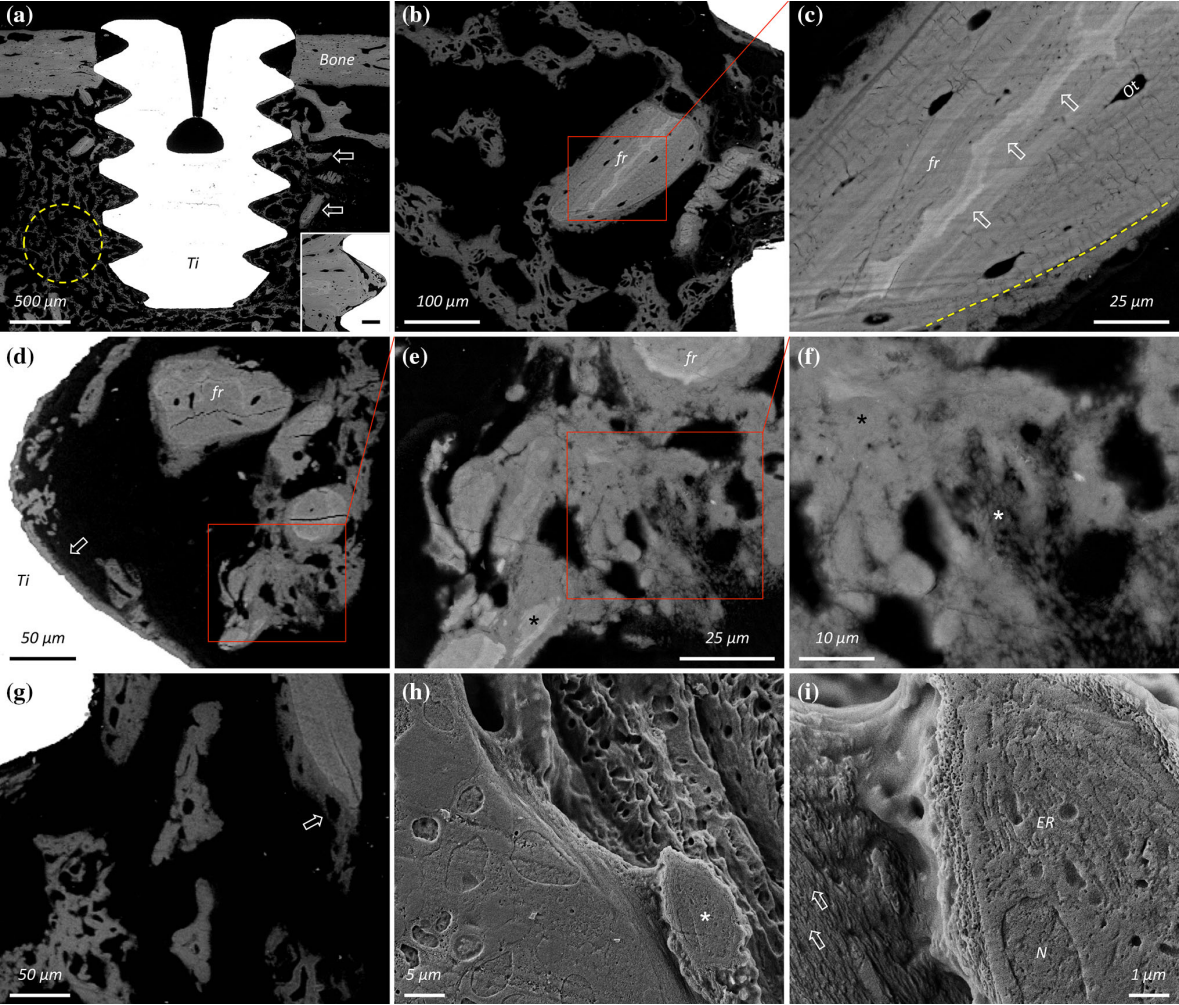
**a** A screw-shaped, machined, cp-Ti implant (Ti) in Sprague Dawley rat tibia. At one week of healing, large amounts of rapidly formed woven bone (*yellow ring*) and autogenous bone fragments of varying dimensions (*arrows*) are observed within and around the implant threads. *Inset:* A similarly prepared implant surface at 28 days of healing (*Scale bar* 100 µm). **b**, **c** Fragments (fr) of the original cortical bone, identified in the rat by the presence of islands of unremodelled hypermineralised cartilage (*arrows*), behave as osteoconductive surfaces that support new bone formation. The interface between the autogenous bone fragment and the newly formed bone on the surface is clearly demarcated (*yellow broken line*) by the striking difference in the BSE *Z*-contrast. **d** In addition to bone apposition directly on the implant surface (*arrow*), autogenous bone fragments and woven bone are seen within the implant thread. **e** Woven bone lacks a well-ordered structure. However, tiny fragments of the original organised, lamellar bone (*black asterisk*) become embedded and incorporated within the new forming tissue. **f** Extracellular matrix at different stages of mineralisation can be distinguished by a variation in the BSE *Z*-contrast, where older and relatively more mature tissue (*black asterisk*) appears brighter than younger tissue (*white asterisk*). **g** New bone formation on a large fragment. **h**, **i** Following resin cast etching, the extracellular matrix deposited next to an osteoblastic-osteocyte (*asterisk*) on the autogenous bone fragment exhibits a well-aligned and ordered arrangement that is maintained up to ‘cellular’ length scales (*arrows*). The endoplasmic reticulum (ER) and nucleus (N) can be identified inside the osteoblastic-osteocyte

**Fig. 2:**
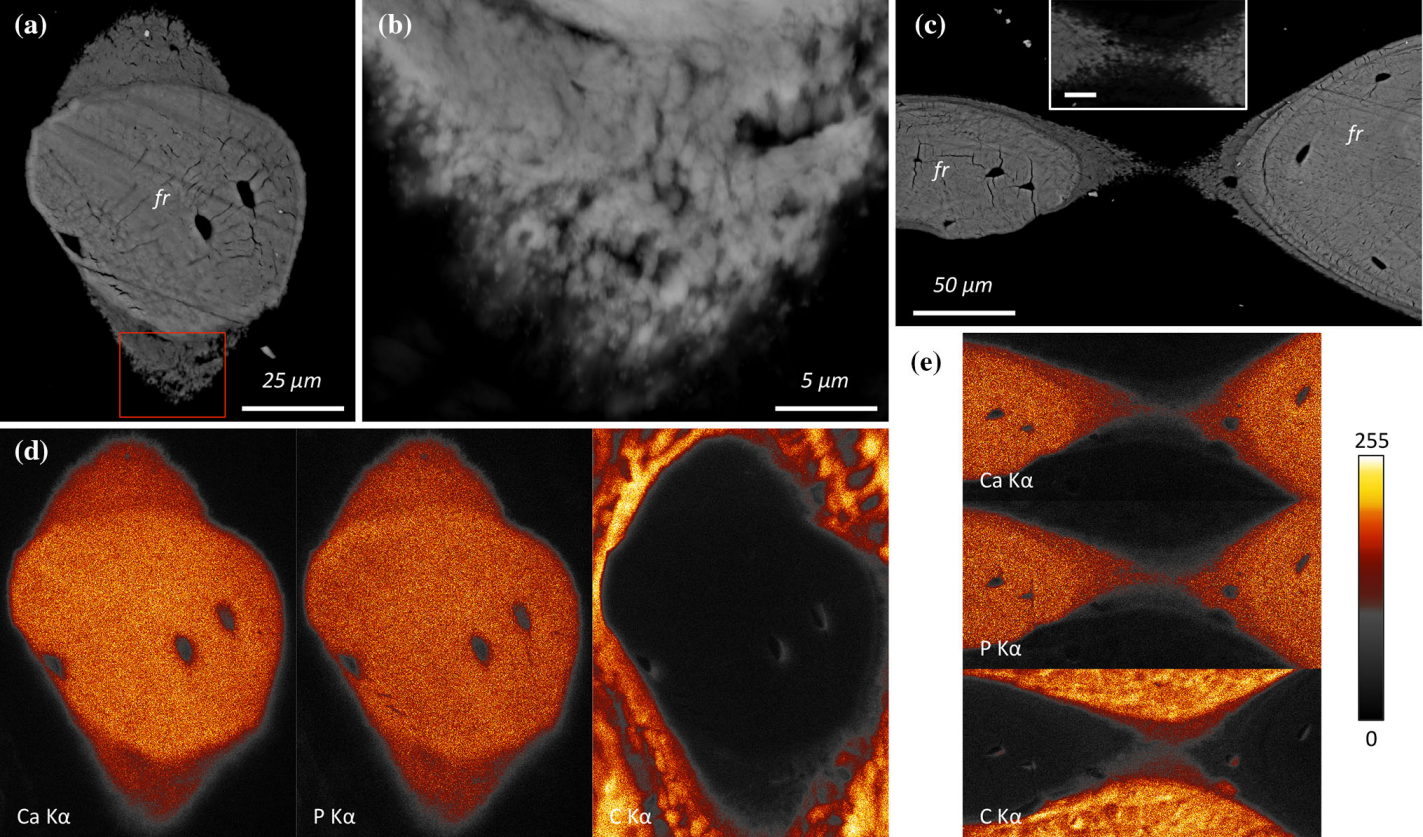
**a**, **b** The mineralisation front appears granular. **c** The newly formed bone bridges the gap between two fragments. *Inset:* Detail of the granular mineralisation front (*Scale bar* 10 µm). **d**, **e** Elemental maps representing the Kα X-ray emission lines for calcium (Ca Kα), phosphorus (P Kα), and carbon (C Kα) corresponding to (**a**) and (**c**), respectively, reveal lower Ca and P content of the newly formed bone compared to the old bone of the fragment

The early healing stage of bone around implanted titanium materials is characterised by the appearance of rapidly formed woven bone (Fig. 1a). Autogenous bone fragments, varying greatly in size and quantity, are frequently observed around the implant and also within the implant threads (Fig. 1b). In the laboratory rat, e.g. Sprague Dawley, fragments of the original cortical bone can be identified by the inclusion of islands of unremodelled hypermineralised cartilage [12]. Such fragments behave as osteoconductive surfaces that support and guide new bone formation (Fig. 1c). Autogenous fragments are also detected in close spatial association with rapidly forming woven bone (Fig. 1d). The extracellular matrix at different stages of mineralisation can be distinguished by a variation in the BSE *Z*- (atomic number) contrast, where younger (or less mature) tissue exhibits a lower *Z*-contrast and appears relatively darker due to lower local calcium content (Fig. 1e, f). In contrast to woven bone, partially embedded osteocytes in the new forming bone, also referred to as osteoblastic-osteocytes [13], are aligned parallel to the surface of the underlying fragment, and subsequently the extracellular matrix deposited on the fragment surface appears well aligned and ordered (Fig. 1g–i). The mineralisation front exhibits a granular appearance due to the presence of sub-micron sized foci (Fig. 2a, b), consistent with the description of mineralised spheres associated with the cellular processes of primary osteoblasts and of MLO-A5 (postosteoblast/preosteocyte-like) cells [14]. When several fragments are located close together, the newly formed bone on their surface may bridge the intervening space (Fig. 2c). Elemental analysis, by EDX, confirms lower calcium and phosphorus content of the newly formed tissue on the fragment surface than the old bone of the fragments (Fig. 2d, e).

**Fig. 3:**
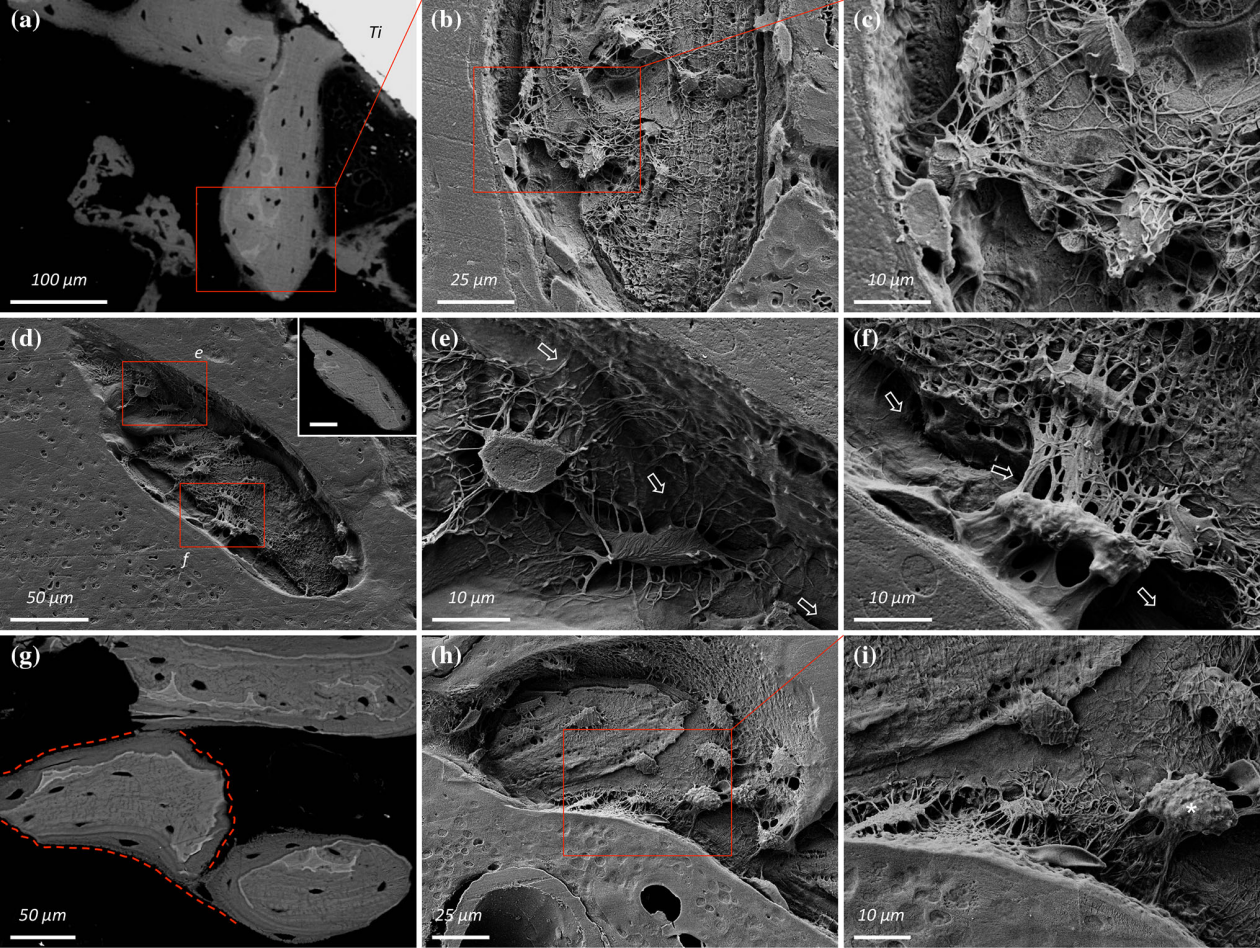
**a** Example #1: A large autogenous bone fragment within an implant thread (Ti) with newly formed bone on the surface. **b**, **c** Following resin cast etching, a high degree of interconnectivity is observed between osteocytes in the newly formed bone and those in the underlying fragment. **d** Example #2: A large autogenous bone fragment outside the implant thread with newly formed bone on the surface. *Inset:* BSE-SEM image of the fragment (*Scale bar* 50 µm). **e**, **f** At regions indicated in (**d**), networks of interconnected resin-filled canaliculi cross the interface (*arrows*) between the old bone of the fragment and the newly formed bone on the surface, and extend up to the marrow space. **g** Example #3: Several autogenous bone fragments in close proximity to each other. **h**, **i** Following resin cast etching, canaliculi from an osteocyte (*asterisk*) in one of the fragments extend towards the marrow space

As bone formation proceeds, osteocytes within the autogenous bone fragments appear to interact physically with osteocytes in the de novo formed bone on the fragment surface. Canaliculi from the osteocytes residing within the old bone of the fragments extend beyond the original periphery and into the new forming tissue where they make numerous connections with newer osteocytes (Fig. 3).

**Fig. 4:**
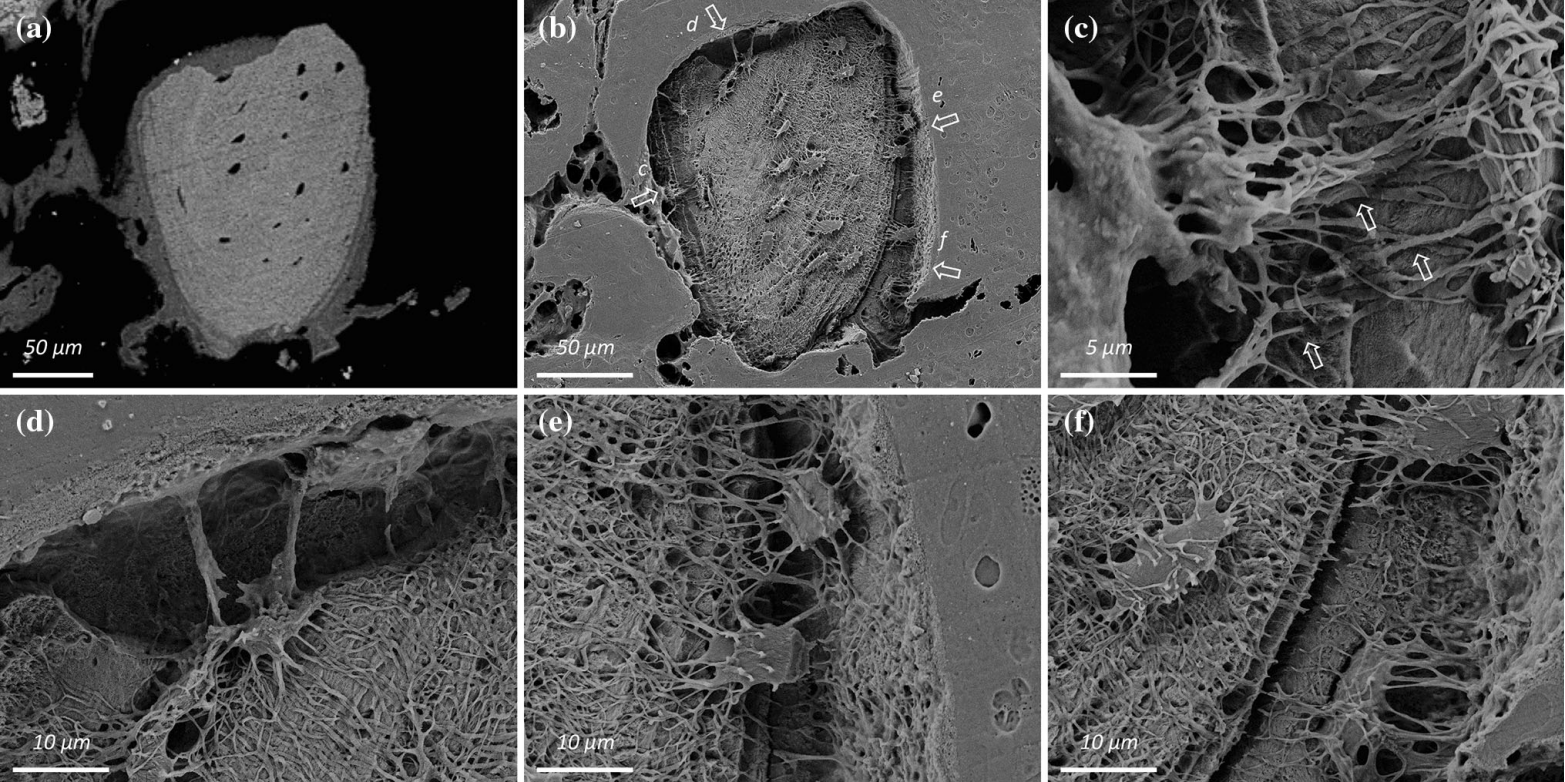
**a** An autogenous bone fragment outside the implant thread that presents a large surface area for new bone formation. The newly formed bone covers approximately 75% of the surface, and occupies an area nearly 38% of the area of the underlying fragment. **b**–**f** Following resin cast etching, at regions indicated in (**b**), osteocytes in the autogenous bone fragment connect with those in the newly formed bone and thereby afford connectivity with the surrounding marrow space

Attributable to the inverse relationship between particle size and surface-to-volume ratio, small autogenous bone fragments present a large surface area for new bone formation (Fig. 4a, b). It is not infrequent to observe high coverage of the fragment’s surface with newly formed bone, and even at this early healing stage, the newly formed bone occupies a large surface area. Moreover, by virtue of a physical interaction between osteocytes within the old bone of the fragments and those in the newly formed bone, osteocytes residing deep within the fragments maintain, or alternatively, regain connectivity with the surrounding marrow space (Fig. 4c–f).

## Discussion and Conclusions

Implant surfaces considered to exhibit a higher osteogenic potential display a higher degree of bone-implant contact or direct bone apposition, often referred to as *contact osteogenesis*. As a consequence of such an osteogenic potential, precursor cells, i.e. mesenchymal stem cells (MSCs) are recruited to an implant (or a surgical defect) site, resulting in high amounts of rapidly formed woven bone. This early-formed tissue, however, lacks a well-ordered structure [15]. Gradually, this tissue is removed by osteoclastic activity and is replaced by organised, lamellar bone—a process that progresses at a considerably slower pace in comparison to the formation of woven bone.

Particulate, decellularised bone has been used as an effective scaffold for bone repair [16]. Small bone fragments in healing bone sites, generated during surgery, lend themselves to a unique scenario where they serve as osteoinductive surfaces and provide attachment sites for bone forming cells, i.e. osteoblasts. It is assumed that canalicular networks associated with the osteocytes nearest to the fragment surface are disrupted and/or destroyed during the surgical procedure. Incorporation of autologous bone grafts occurs through a process termed *creeping substitution* (a slow, near-complete resorption of the graft with simultaneous deposition of new, viable bone) [17]. Contrary to the fate of cortical autografts, where mature osteocytes degenerate in the early stages following transplantation [18], when autogenous bone fragments are generated in the defect during the drilling process without exposure to an ex vivo environment, the osteocytes possibly remain viable and functional.

Here we demonstrate, using correlative scanning electron microscopy (SEM) techniques, that (*i*) autogenous bone fragments contribute towards osteogenesis within healing surgical defects, e.g. in the vicinity of bone-anchored implants, and (*ii*) osteocytes within autogenous bone fragments are frequently observed to restore a close physical proximity with osteocytes (osteoblastic-osteocytes) in new bone formed on the surface of these fragments, through interconnecting canaliculi that contain cytoplasmic extensions of osteocytes. However, it is not known whether the restored interconnectivity between osteocyte canaliculi in old and new bone plays any role in transmitting biochemical signals or transporting biomolecules involved in osteocyte function. Although certain implant surfaces are believed to exhibit an enhanced osteogenic potential and stronger mechanical interlocking with the surrounding bone tissue [19, 20], it is assumed that the presence of autogenous bone fragments within a healing surgical defect, and the prevalence thereof, is a function of the surgical technique. Therefore, the physico-chemical properties of the implant surface have no direct bearing on the osteopromotive potential of such bone fragments. Nevertheless, bone drilling may induce osteocyte death, particularly as a function of time [21], and temperature [22].

Further experiments are required to ascertain the viability and the eventual fate of the osteocytes in autogenous bone fragments. Such information may be of benefit in optimising surgical and drilling techniques in order to minimise their destructive effects on the implantation site. The presence of bone fragments at experimental implantation sites, particularly within implant threads, and their osteogenic potential have wide-ranging implications on peri-implant healing. It may be appreciated that, at least during early healing, such autogenous bone fragments (and the associated de novo formed bone) contribute to the overall amount of mineralised tissue found within the healing defect in addition to bone apposition directly on the implant surface.
